# Supplementary material for: A dual role of prestimulus spontaneous neural activity in visual object recognition
Source: Nat Commun. 2019 Sep 2;10:3910. doi: 10.1038/s41467-019-11877-4 (PMC6718405; doi:10.1038/s41467-019-11877-4)
Supplement: Supplementary file 1 — Supplementary Information [file 41467_2019_11877_MOESM1_ESM.pdf]

A dual role of prestimulus spontaneous neural activity in visual object recognition

Podvalny et al.

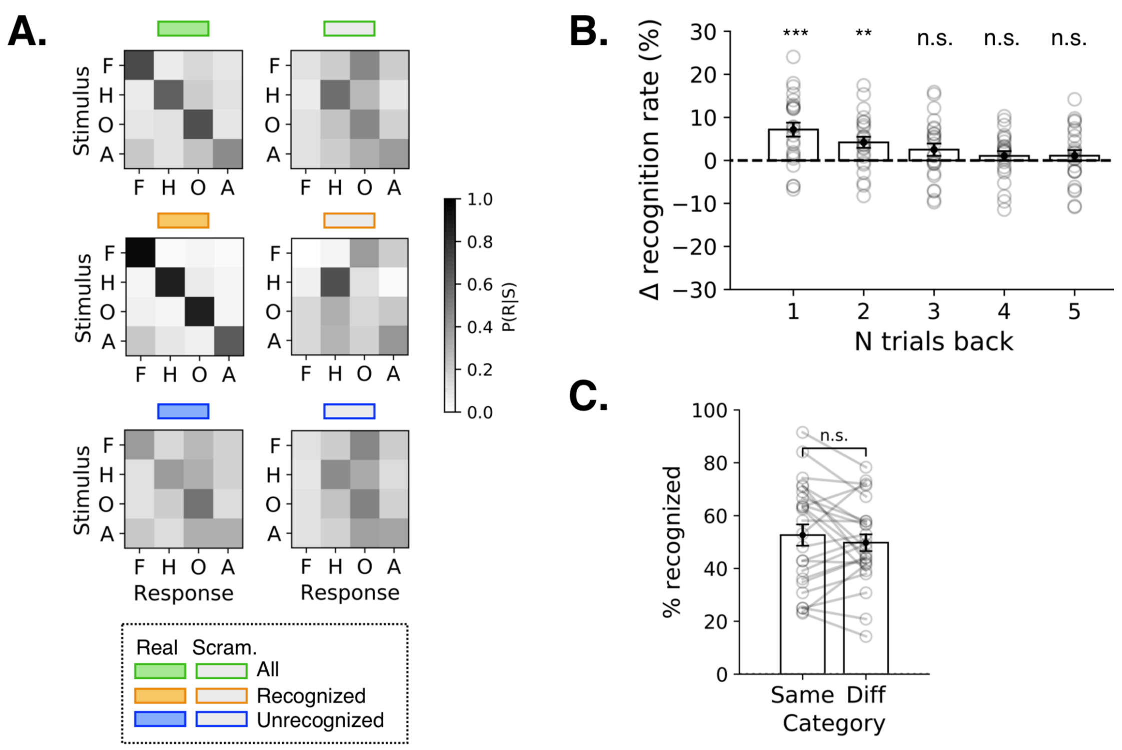

Supplementary Figure 1. Additional behavioral results. A. Categorization confusion matrices presenting probability of reported stimulus category (x axis) given objective stimulus category (y axis). **B.** Influence of a subjective recognition report ("yes" vs. "no") on previous trials on the subjective recognition rate. Δ recognition rate (%) is calculated as a difference in recognition rate preceded by a "yes" trial and a "no" trial and tested for significance using Wilcoxon sign-ranked test (N=24). **C.** Influence of objective stimulus category on a previous trial (same as the category on a current trial or different) on subjective recognition rate (Wilcoxon sign-ranked test, N=24).

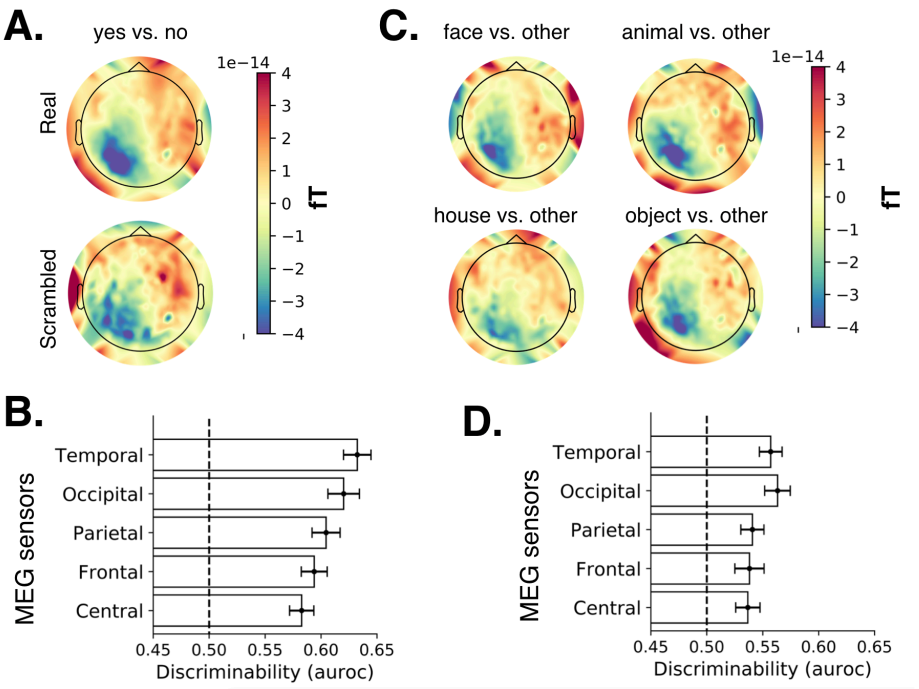

Supplementary Figure 2. Spatial correlates of pre-stimulus activity patterns influencing object recognition. (**A**) Activation patterns[1] of a logistic regression model, decoding recognition experience (yes vs. no) using pre-stimulus activity patterns in real-image trials (N = 24). (**B**) Discriminability (AUROC) of recognized vs. non-recognized trials for logistic regression models fitted using separate groups of MEG sensors. There is a significant effect of sensor group on model performance, whereby sensors overlying occipitotemporal brain regions provided maximal AUROC values (repeated measures ANOVA, $F(4, 23) = 4.84$, $p = 10^{-3}$) **(C)** Same as A for a model decoding objective stimulus category using pre-stimulus activity patterns in recognized real-image trials. Activation patterns are calculated using decoder weights discriminating one category versus others. (**D**) Same as B for stimulus category decoding. There is a significant effect of sensor group on model performance (repeated measures ANOVA, $F(4, 23) = 2.71$, $p = 0.03$), whereby sensors overlying occipitotemporal brain regions provided maximal AUROC values.

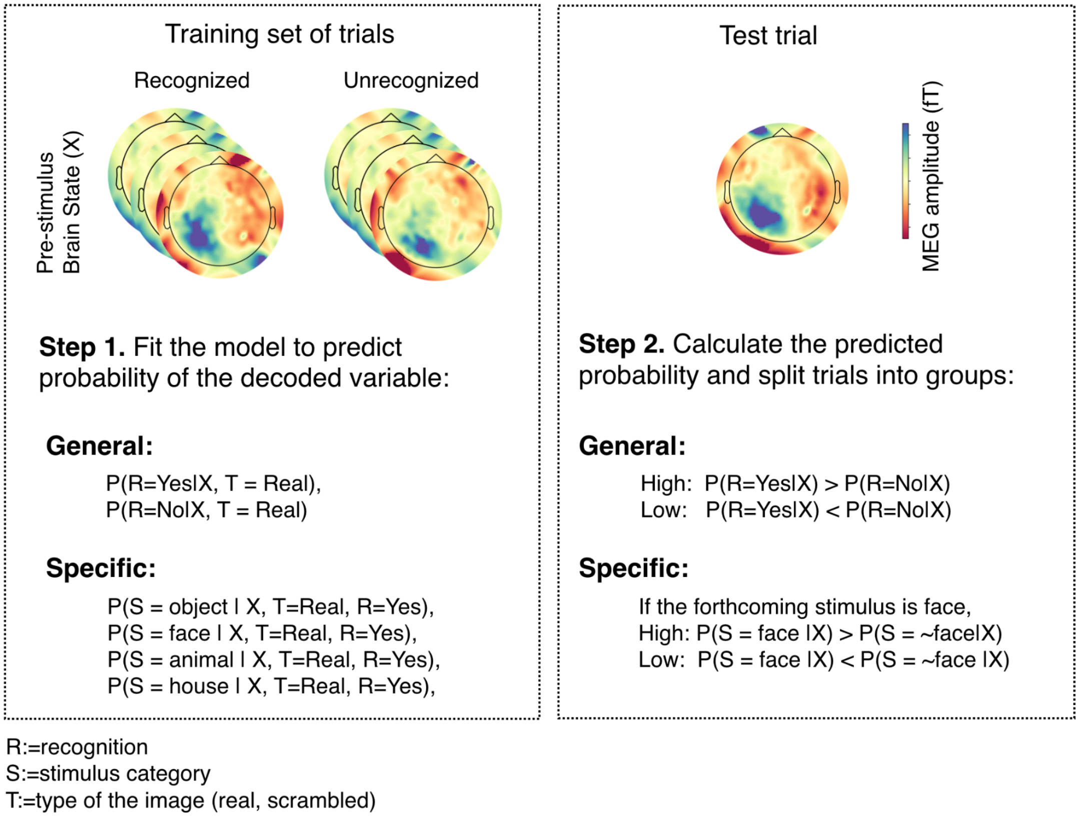

Supplementary Figure 3. Schematics of probability groups split according to the General and Specific models. Step 1: We fit the weights of the logistic regression decoder using a training set of trials. For the General model we used real-image trials to decode the recognition report (yes/no). For the Specific model we used real-image trials that were recognized to decode the stimulus category. **Step 2**: Using the fitted logistic regression decoder from step 1, we calculate the probability of the decoded variable for each single test trial. For the General model, the trial will be placed in the high-probability group if the logistic regression model predicts that it is more likely to be recognized than unrecognized. For the Specific model, the trial will be placed in the high-probability group if the logistic regression model predicts the forthcoming stimulus category with a probability that is higher than that of every other category. Note, there is no overlap between trials used in the two steps.

Supplementary references

1. Haufe, S. *et al.* On the interpretation of weight vectors of linear models in multivariate neuroimaging. *Neuroimage* **87**, 96–110 (2014).
