# Supplementary material for: A dual role of prestimulus spontaneous neural activity in visual object recognition
Source: Nat Commun. 2019 Sep 2;10:3910. doi: 10.1038/s41467-019-11877-4 (PMC6718405; doi:10.1038/s41467-019-11877-4)
Supplement: Supplementary file 2 — Description of Additional Supplementary Files [file 41467_2019_11877_MOESM2_ESM.docx]

Description of Additional Supplementary Files

**Supplementary Code 1:** Provides details on how we used the sklearn toolbox pipelines for the multivariate analyses.
